# Exposure of Primary Human Skin Fibroblasts to Carbon Dioxide-Containing Solution Significantly Reduces TGF-β-Induced Myofibroblast Differentiation In Vitro

**DOI:** 10.3390/ijms252313013

**Published:** 2024-12-03

**Authors:** Maxine Fleckner, Niklas K. Döhmen, Katharina Salz, Till Christophers, Joachim Windolf, Christoph V. Suschek, Lisa Oezel

**Affiliations:** Department for Orthopedics and Trauma Surgery, Medical Faculty and University Hospital Düsseldorf, Heinrich Heine University Düsseldorf, 40225 Dusseldorf, Germany

**Keywords:** myofibroblast differentiation, carbon dioxide (CO_2_), alpha-smooth muscle actin (α-SMA), glycolysis, mitochondrial respiration, cellular energy metabolism

## Abstract

Wound healing as a result of a skin injury involves a series of dynamic physiological processes, leading to wound closure, re-epithelialization, and the remodeling of the extracellular matrix (ECM). The primary scar formed by the new ECM never fully regains the original tissue’s strength or flexibility. Moreover, in some cases, due to dysregulated fibroblast activity, proliferation, and differentiation, the normal scarring can be replaced by pathological fibrotic tissue, leading to hypertrophic scars or keloids. These disorders can cause significant physical impairment and psychological stress and represent significant challenges in medical management in the wound-healing process. The present study aimed to investigate the therapeutic effects of exogenously applied carbon dioxide (CO_2_) on fibroblast behavior, focusing on viability, proliferation, migration, and differentiation to myofibroblasts. We found that CO_2_ exposure for up to 60 min did not significantly affect fibroblast viability, apoptosis rate, or proliferation and migration capacities. However, a notable finding was the significant reduction in α-smooth muscle actin (α-SMA) protein expression, indicative of myofibroblast differentiation inhibition, following CO_2_ exposure. This effect was specific to CO_2_ and concentration as well as time-dependent, with longer exposure durations leading to greater reductions in α-SMA expression. Furthermore, the inhibition of myofibroblast differentiation correlated with a statistically significantly reduced glycolytic and mitochondrial energy metabolism, and as a result, with a reduced ATP synthesis rate. This very noticeable decrease in cellular energy levels seemed to be specific to CO_2_ exposure and could not be observed in the control cultures using nitrogen (N_2_)-saturated solutions, indicating a unique and hypoxia-independent effect of CO_2_ on fibroblast metabolism. These findings suggest that exogenously applied CO_2_ may possess fibroblast differentiation-reducing properties by modulating fibroblast’s energy metabolism and could offer new therapeutic options in the prevention of scar and keloid development.

## 1. Introduction

Fibroproliferative and sclerotic diseases, e.g., Dupuytren’s disease, as well as excessive scar tissue formation are restraining disorders with a high impact on human health. In terms of excessive scar formation, tissue remodeling processes and mechanisms often show a disorder. Hereby, fibrogenesis and certainly myofibroblast differentiation seem to play a crucial role. Although fibrotic disorders distinguish in etiological and clinical features, they are all based on an excessive production of growth factors, proteolytic enzymes, and angiogenic as well as fibrogenic factors and cytokines [1,2]. The interaction of all these factors leads to an increased differentiation of fibroblasts to myofibroblasts and thus to fibroproliferative processes that stimulate and remodel tissue architecture.

Myofibroblasts have diverse origins; myofibroblasts may derive from local mesenchymal cells, may be transformed epithelial cells, or may derive from circulating mesenchymal stem cells originating in the bone marrow [3]. They are characterized by enhanced contractility mediated by an expression of stress fibers known as α-smooth muscle actin (α-SMA) [2,4]. Following activation, myofibroblasts coordinate the repair and remodeling of connective tissue. Any kind of excessive activity of myofibroblasts can lead to pathological tissue formations, resulting in fibrosis or pronounced scar tissue. Indicative of all sclerotic diseases is a high immigration and proliferation rate of fibroblasts as well as an increased either spontaneous or transforming growth factor-β (TGF-β)-induced differentiation of fibroblasts to myofibroblasts. These mechanisms are accompanied by a high production of collagen and reticular fibers and a decreased apoptosis rate of fibroblasts and myofibroblasts in affected tissues. TGF-β, with its indispensable function in growth and tissue remodeling, triggers fibroblast proliferation and differentiation into myofibroblasts [5]. Thus, the increased expression of TGF-β is a main factor of fibrotic diseases. There are different pathways proven to play a role in the activation and modulation of TGF-β expression, e.g., reactive oxygen species (ROS) [6,7]. In this context, TGF-β leads to an increased ROS production, which in turn leads to an impaired mitochondrial function and further to an induction of NADPH oxidases (NOXs) [6,8]. Moreover, TGF-β is known for the suppression of antioxidants, thereby causing a redox imbalance leading to oxidative stress. Redox imbalance further induces TGF-β, which reveals the autocrine and paracrine mechanisms inducing TGF-β-mediated fibrogenic activity [9].

Relevant approaches in the therapy of sclerotic skin disorders have been X-ray, visible light [10,11,12], intralesional and local application of cortisone or immunosuppressants [13], laser-therapy, cryotherapy [14], and surgical removal [15]. As the main issue of these approaches is a high recurrence rate, there is still the need for effective, new therapies but primarily for effective preventive measures. Scar prevention is a critical aspect of wound management, with significant implications for functional, cosmetic, and psychological outcomes. Current evidence highlights the efficacy of silicone-based products, such as gels and sheets, as first-line therapies for scar prevention due to their ease of use and proven benefits, particularly for high-risk populations prone to keloid formation [16]. Advances in understanding the wound-healing process have identified key phases, such as inflammation, epithelial–mesenchymal transition, and myofibroblast activity, as critical targets for preventing fibrosis, with multimodal approaches showing promise [17]. Corticosteroids remain standard treatments for keloids and hypertrophic scars, though their broader role in scar prevention requires further exploration [18]. Molecular mediators, such as the inhibitors of the IL-4/IL-13 axis and TGF-β pathways, have also emerged as promising strategies to reduce fibrotic activity [19]. Mechanotherapy, which involves modulating mechanical forces during wound healing, has demonstrated potential for optimizing scar outcomes when combined with pressure therapies and tension-offloading approaches [20]. Emerging therapeutics, such as transforming growth factor-beta 3 (TGF-β3), offer promise for scar reduction by promoting skin regeneration, though more comprehensive trials are necessary to validate these approaches [21]. Additionally, the early control of inflammation, inspired by fetal wound-healing processes that naturally avoid scarring, has been identified as a crucial intervention to improve scar outcomes [22].

With this regard, the application of carbon dioxide (CO_2_) could present an alternative solution, as it has already in the past been known for its therapeutic and beneficial effects on microcirculation and tissue oxygenation. In 2011, Vohwinkel et al. demonstrated that increased levels of CO_2_ impair cell proliferation and therefore have positive effects in patients with lung fibrosis [23]. In the study of Brandi et al., the subcutaneous application of CO_2_ showed progress in the healing of chronic wounds [24]. Moreover, Finzgar et al. stated in their study the vasodilatory effect of transcutaneously applied CO_2_ therapy [25]. Other studies, in general, have shown positive therapeutic effects of the trans- or subcutaneous application of CO_2,_ including reduced fluctuations in blood pressure (calming effect) and increased oxygenation, blood flow, and blood vessels in ischemic limbs [26,27,28]. Moreover, the transcutaneous application of CO_2_ induces the expression of the receptors involved in mitochondrial biogenesis and increases mitochondrial proliferation and apoptosis in human malignant fibrous histiocytoma cells in vitro [29]. Further on, previous studies demonstrated that the transcutaneous application of CO_2_ increases the number of muscle mitochondria and promotes muscle endurance in a rat model [30].

In the present study, we have investigated the impact of CO_2_-saturated aqueous solutions and culture media on the fibrosis-relevant parameters of human skin fibroblasts, like viability, proliferation, migration capacity, and spontaneous and TGF-β-induced myofibroblast differentiation.

## 2. Results

### 2.1. Impact of CO_2_ or N_2_ Exposure on Viability, Proliferation, and Migration of Primary Human Skin Fibroblasts

The primary cultures of the human skin fibroblasts were incubated with CO_2_-saturated solutions for up to 60 min or, alternatively, maintained for 30 min in a CO_2_- or N_2_-flooded hyperbaric chamber. Subsequently, after 3 or 24 h, the vitality of the cells was quantified using the CellTiter-Blue assay, the apoptosis rate was carried out using the FACS-supported Nicoletti test, and the proportion of secondary necrosis in the gas-exposed cultures was determined using propidium iodide and fluorescence microscopy.

**Figure 1:**
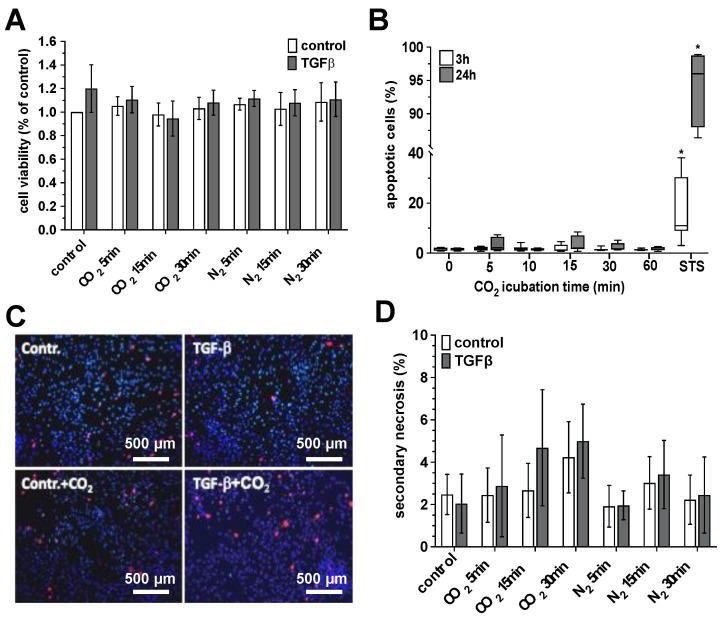
Impact of CO_2_- or N_2_-saturated solutions on viability of primary human skin fibroblasts. The primary cultures of the human skin fibroblasts were incubated with CO_2_- or N_2_-saturated solutions for the time intervals indicated. After 24 h, the vitality of the cells was quantified using the cell titer blue assay (**A**), the apoptosis rate was carried out by FACS analysis (**B**), and the proportion of secondary necrosis was determined using propidium iodide and fluorescence microscopy (**C**,**D**). (**A**), Vitality rates of the untreated (white bars) and TGF-β-activated (gray bars) skin fibroblasts after exposure to CO_2_- or N_2_-saturated solutions. Bars represent the mean ± S.D. of eight individual experiments (*n* = 8). (**B**), Rates of apoptotic events in the skin fibroblast cultures 3 h (white boxes) and 24 h (gray boxes) after exposure to CO_2_- or N_2_-saturated solutions or the apoptosis-inducing agent staurosporine (STS, 1 µg/mL). The values of 5 individual (*n* = 5) experiments are shown as boxplots with median values and with whiskers with minimum and maximum. *, *p* < 0.05 as compared to the non-treated cultures (0 min CO_2_ incubation). (**C**), Photomicrographs of representative fluorescence microscopy-based investigation of the control, TGF-β-activated, and CO_2_-exposed (30 min with CO_2_-saturated medium) skin fibroblast cultures stained with the Hoechst 33342 dye (nuclei) and propidium iodide (necrotic cells, secondary necrosis). (**D**), Quantitative evaluation of the cells exhibiting signs of secondary necrosis (propidium-positive cells) in the untreated (white bars) and TGF-β-activated (gray bars) skin fibroblast cultures 24 h after a 30 min exposition to with CO_2_- or N_2_-saturated media. Bars represent the mean ± S.D. of eight individual experiments (*n* = 8).

As we show in Figure 1, the gas exposure procedure described above did not result in any significant change in the viability, apoptosis rate, or secondary necrosis formation of the treated cultures compared to the untreated cultures.

In addition, we evaluated the influence of daily 30-min exposure to saturated carbonated media for three days on the proliferation rate and characterized the impact of a single 30-min CO_2_ treatment on the migration rate of the exposed fibroblast cultures.

**Figure 2:**
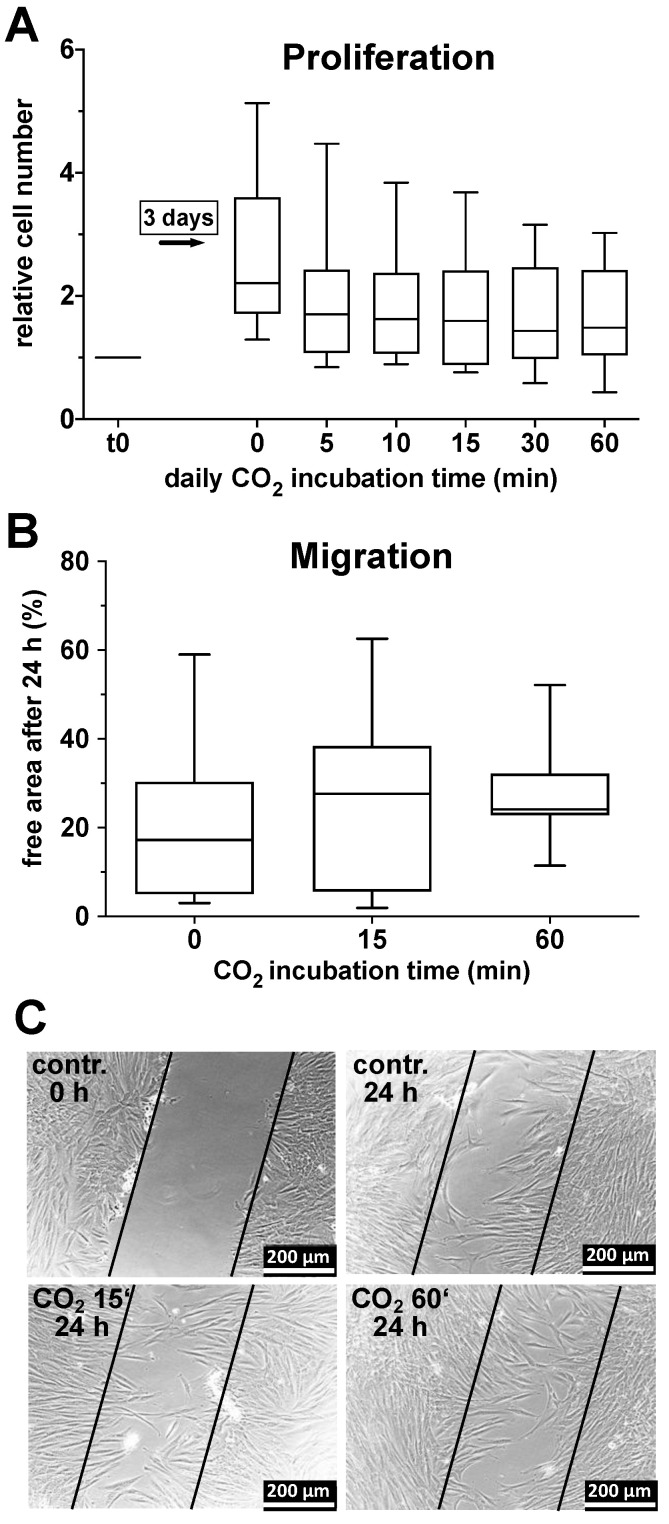
Impact of CO_2_-saturated solutions on proliferation and migration of primary human skin fibroblasts. (**A**), Fibroblast proliferation: Primary human skin fibroblasts (1 × 10^4^ cells/well at time point t_0_) grown in 24-well cell culture plates were incubated at day 1, 2, and 3 for 5, 10, 15, 30, or 60 min each with CO_2_-saturated DMEM. The cell number of the respective cultures was detected by the CellTiter-Blue assay on day 4. The values of 8 individual (*n* = 8) experiments are shown as boxplots with median values and with whiskers with minimum and maximum. (**B**), Fibroblast migration (“scratch assay”): After creating a “scratch” in a fibroblast monolayer and defining the area of the scratch as 100%, the remaining cell-free area, given in %, was detected 24 h after scratching in the control as well as the cultures exposed to CO_2_-saturated solutions for 15 or 60 min prior the test. The values of 3 individual (*n* = 3) experiments are shown as boxplots with median values and whiskers representing minimum and maximum. (**C**), Representative photomicrographs of one individual experiment as described in (**B**).

The results of these series of tests, graphically evaluated in Figure 2, demonstrate that the experimental procedure did not lead to a statistically significant change in the proliferation and migration rate of the fibroblast cultures compared to the control cultures.

### 2.2. Impact of CO_2_- and N_2_-Saturated Solutions on α-SMA Protein Expression of Primary Human Skin Fibroblasts

The TGF-β-activated fibroblast cultures were treated with carbonized solutions daily during the three-day differentiation phase. The CO_2_ exposure period (1–60 min) or the level of carbonization of the solution (20%, 40%, 60%, 80%, and 100% of maximal saturation) was varied. We reached the aforementioned CO_2_ concentration gradients of the solutions by diluting CO_2_-saturated solutions with non-carbonized solutions. Untreated or N_2_-saturated solutions were used as control. The relative protein expression of the alpha-smooth muscle actin (α-SMA) was quantified as a qualitative parameter of fibroblast differentiation 24 h after the last treatment.

**Figure 3:**
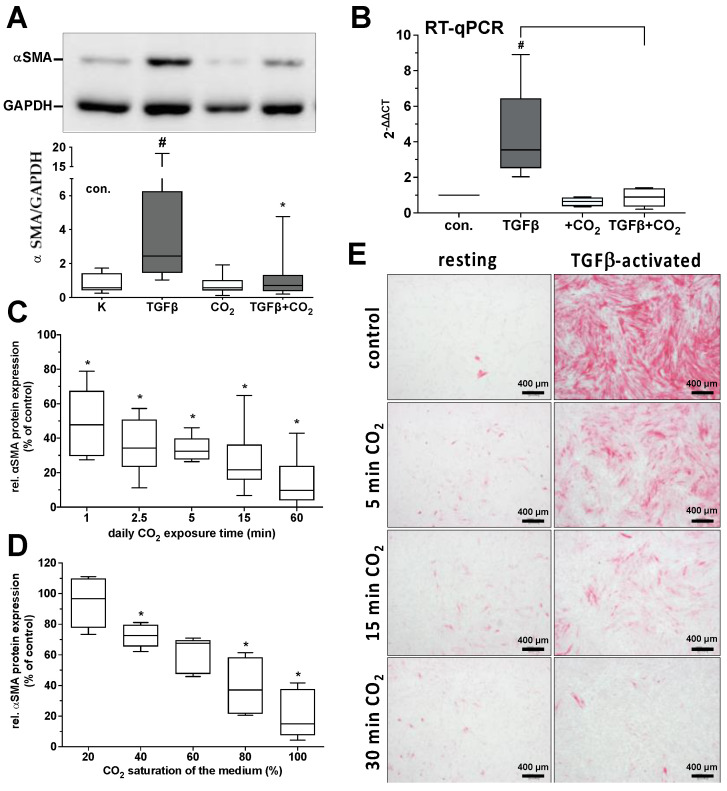
Impact of CO_2_-saturated solutions on the α-SMA protein expression of primary human skin fibroblasts. The untreated or TGF-β (2 ng/mL)-activated fibroblast cultures were treated for three days daily with carbonized solutions (20%, 40%, 60%, 80%, and 100% of maximal saturation) for 1, 2.5, 5, 15, 30, or 60 min. Untreated solutions were used as control. The relative protein expression of the alpha-smooth muscle actin (α-SMA) protein (**A**) and mRNA expression (**B**) was quantified by Western blot and RT-qPCR as the qualitative parameters of fibroblast differentiation 24 h after the last treatment. (**A**), α-SMA protein expression and (**B**), mRNA expression after daily 30 min treatment with saturated carbonized solutions. In (**A**), the values of 8 individual experiments (*n* = 8) and in (**B**) of 6 individual experiments (*n* = 6) are shown as boxplots with median values and whiskers representing minimum and maximum. #, *p* < 0.05 as compared to the untreated control cultures. *, *p* < 0.05 as compared to the respective TGF-β-activated cultures maintained in the absence of CO_2_. (**C**), Relative TGF-β-activated α-SMA protein expression after daily treatment with saturated carbonized solutions for the time periods indicated. The values of 8 individual experiments (*n* = 8) are shown as boxplots with median values and whiskers representing minimum and maximum. *, *p* < 0.05 as compared to the untreated control cultures and TGF-β-activated fibroblast cultures maintained in the absence of CO_2_. (**D**), Relative TGF-β-activated α-SMA protein expression following daily treatment (15 min) with carbonized solutions of varying CO_2_ saturation levels. The values of 8 individual experiments (*n* = 8) are shown as boxplots with median values and whiskers representing minimum and maximum. *, *p* < 0.05 as compared to TGF-β-activated fibroblast cultures treated with 100% saturated CO_2_ solutions. (**E**), Exemplary photographs of an individual experiment for the immunocytochemical visualization of α-SMA expression (red signal) in the untreated and TGF-β-activated fibroblast cultures after treatment with CO_2_-saturated solutions at the indicated exposure times.

Already, a daily one-minute CO_2_ exposure of the TGF-β-activated fibroblast cultures over three days led to a statistically significant reduction in the α-SMA protein expression (Figure 3B). With longer exposure times, the degree of reduction in the α-SMA protein expression increased, reaching an 80% reduction compared to the untreated cultures (Figure 3A) at a treatment time of 60 min.

Furthermore, we achieved an analogous pattern of reduction in α-SMA protein expression by treating the fibroblasts with solutions with different degrees of carbonization. With a 40% saturated CO_2_ solution, we observed a statistically significant decrease in α-SMA protein expression by approx. 30% (Figure 3C); with a 60% saturated solution by approx. 40%; and with an 80% saturated solution by approx. 60% (Figure 3C).

**Figure 4:**
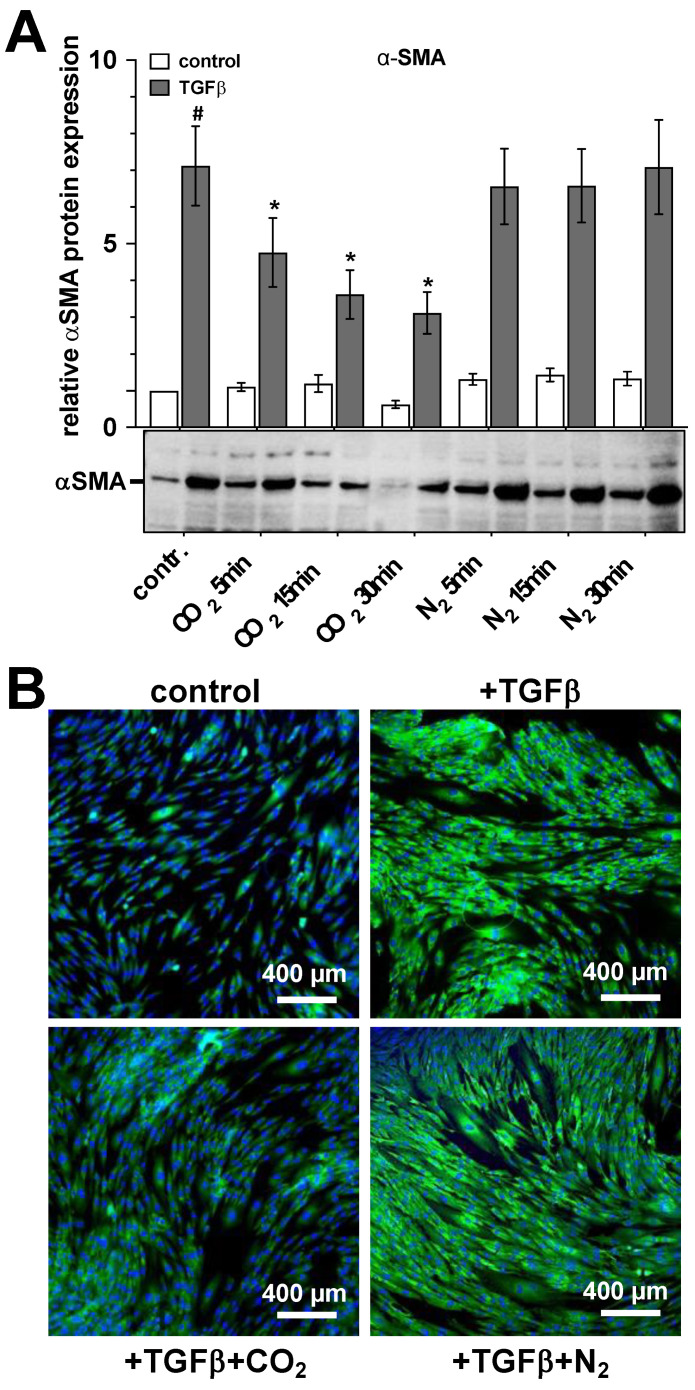
Comparison of the impacts of CO_2_- or N_2_-saturated solutions on the α-SMA protein expression of primary human skin fibroblasts. The untreated or TGF-β (2 ng/mL)-activated fibroblast cultures were treated for three days daily with CO_2_- or N_2_-saturated solutions for the time intervals indicated. The relative protein expression of the alpha-smooth muscle actin (α-SMA) protein expression was quantified by Western blot as a qualitative parameter of fibroblast differentiation 24 h after the last treatment. (**A**), Bars represent the mean ± S.D. of the relative α-SMA expression in the untreated (white bars) and TGF-β-activated (gray bars) human skin fibroblasts of 8 individual experiments (*n* = 8). #, *p* < 0.05 as compared to the control (contr.); *, *p* < 0.05 as compared to the TGF-β-activated cell cultures maintained in the absence of CO_2_. (**B**), Exemplary photographs of an individual experiment for the immunocytochemical visualization of α-SMA expression (green fluorescent signal) in the untreated and TGF-β-activated fibroblast cultures after a 30-min treatment with CO_2_- or N_2_-saturated solutions. The blue signal represents nuclear staining using the Hoechst fluorescent dye.

The last discussed α-SMA protein expression-reducing effect of CO_2_ treatment seems to represent a CO_2_-specific mechanism as an identical treatment procedure, but the use of N_2_ gas instead of CO_2_ did not lead to any statistically significant alterations in α-SMA protein expression (Figure 4A,B).

**Figure 5:**
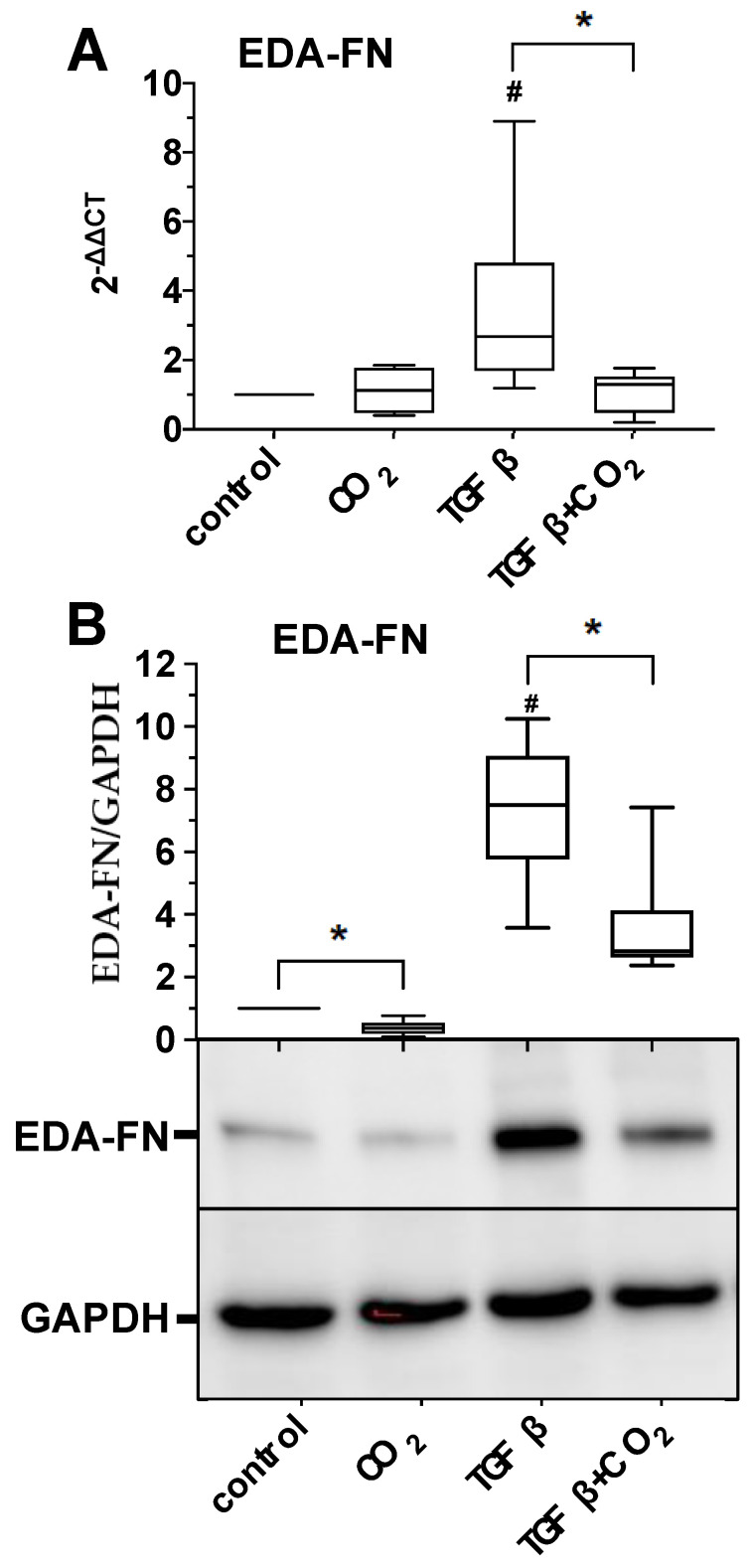
Impact of CO_2_-saturated solutions on the EDA-FN mRNA and protein expression of primary human skin fibroblasts. The untreated or TGF-β (2 ng/mL)-activated fibroblast cultures were treated for three days daily with CO_2_-saturated solutions for 30 min (gray bars). Untreated solutions were used as control. The relative mRNA (**A**) and protein expression (**B**) of fibronectin extracellular domain A (EDA-FN) were quantified by real-time PCR (**A**) or Western blot technique (**B**). In (**A**,**B**), the values of 5 individual experiments (*n* = 5) are shown as boxplots with median values and whiskers representing minimum and maximum. #, *p* < 0.05 as compared to the untreated control cultures maintained in the absence of CO_2_. *, *p* < 0.05.

To further support our assumption of an antifibrotic effect of CO_2_, we characterized the impact of CO_2_-saturated buffer solutions on the TGF-β-induced expression of the fibronectin extra domain A (EDA-FN), another molecular key parameter of myofibrogenesis. The results of these experimental series, as shown in Figure 5, clearly demonstrate that CO_2_ causes a significant reduction both at the gene expression level and at the protein expression level, even in the case of EDA-FN.

**Figure 6:**
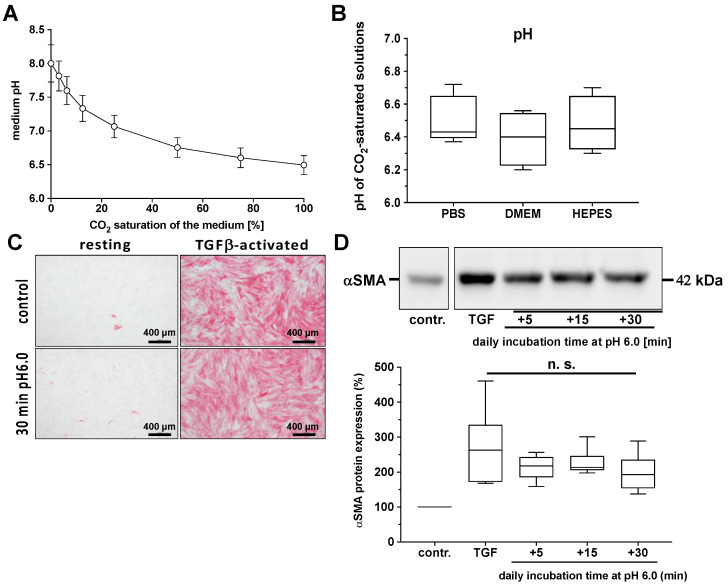
Impact of low pH on the α-SMA protein expression of primary human skin fibroblasts. (**A**), pH values of non-buffered DMEM with varying levels of CO_2_ saturation. (**B**), pH values of CO_2_-saturated buffered solutions, PBS, DMEM, and DMEM + HEPES. The values of four individual experiments (*n* = 4) are shown as boxplots with median values and whiskers representing minimum and maximum. Additionally, the untreated or TGF-β (2 ng/mL)-activated fibroblast cultures were treated for three days daily with a medium at pH 6.0 for the time intervals indicated. The relative protein expression of the alpha-smooth muscle actin (α-SMA) protein expression was quantified by Western blot as a qualitative parameter of fibroblast differentiation 24 h after the last treatment. (**C**), Exemplary photographs of an individual experiment for the immunocytochemical visualization of α-SMA expression (red signal) in the untreated and TGF-β-activated fibroblast cultures after treatment with the acidified solutions (pH 6.0) for 30 min. (**D**), Relative TGF-β-activated α-SMA protein expression after daily treatment with the acidified solutions for 5, 15, or 30 min, respectively. The values of four individual experiments (*n* = 4) are shown as boxplots with median values and whiskers representing minimum and maximum. n.s., statistically not significant as compared to solely TGF-β-activated fibroblast cultures.

Since the saturating carbonization of solutions led to a significant reduction in the pH value, regardless of the buffer system used (Figure 6A,B), we evaluated the impact of reduced pH on the α-SMA protein expression of TGF-β-activated human fibroblast cultures.

In Figure 6C,D, we show that acidified solutions per se, in the absence of CO_2_, did not significantly affect TGF-β-induced fibroblast differentiation.

### 2.3. Impact of 2-Deoxy-Glucose on TGF-β-Induced α-SMA Protein Expression and Basal ATP Production Rates

**Figure 7:**
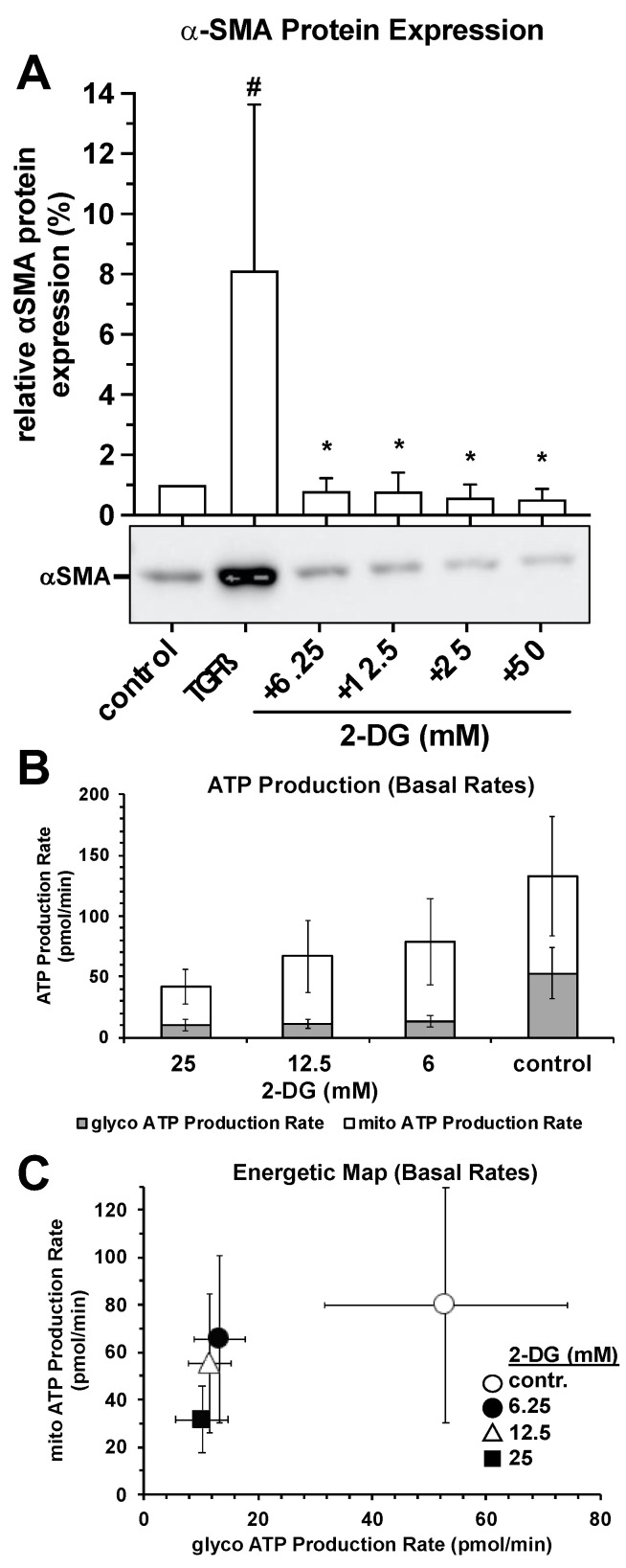
Impact of 2-deoxy-glucose on TGF-β-induced α-SMA protein expression and basal ATP production rates. (**A**), Impact of the glucose analog 2-deoxy-glucose, an effective non-competitive hexokinase inhibitor, present during the 72-h incubation period, on TGF-β-induced α-SMA protein expression. #, *p* < 0.05 as compared to the untreated control cultures. *, *p* < 0.05 as compared to the respective TGF-β-induced cultures maintained in the absence of 2-DG. (**B**,**C**), The anti-myofibrogenic effect of 2-DG correlated with a strong and statistically significant decrease in basal glycolytic and mitochondrial ATP production rates, which were characterized by the detection of the proton efflux rate (PER) in the cell cultures using the *Agilent Seahorse XF24* system.

The presence of the glucose analog 2-deoxy-glucose, an effective non-competitive hexokinase inhibitor, during the 72-h incubation period led to a complete inhibition of TGF-β-induced α-SMA protein expression already with the lowest 2-DG concentration of 6.25 mM (Figure 7A). The anti-myofibrogenic effect of 2-DG correlated with a strong and statistically significant decrease in basal glycolytic and mitochondrial ATP production rates (Figure 7B,C), which were characterized by the detection of the proton efflux rate (PER) in the cell cultures using the *Agilent Seahorse XF24* system.

### 2.4. Impact of CO_2_- or N_2_-Saturated Solutions, Acidic Solutions, and Lactate on Parameters of Cellular Energy Metabolism

**Figure 8:**
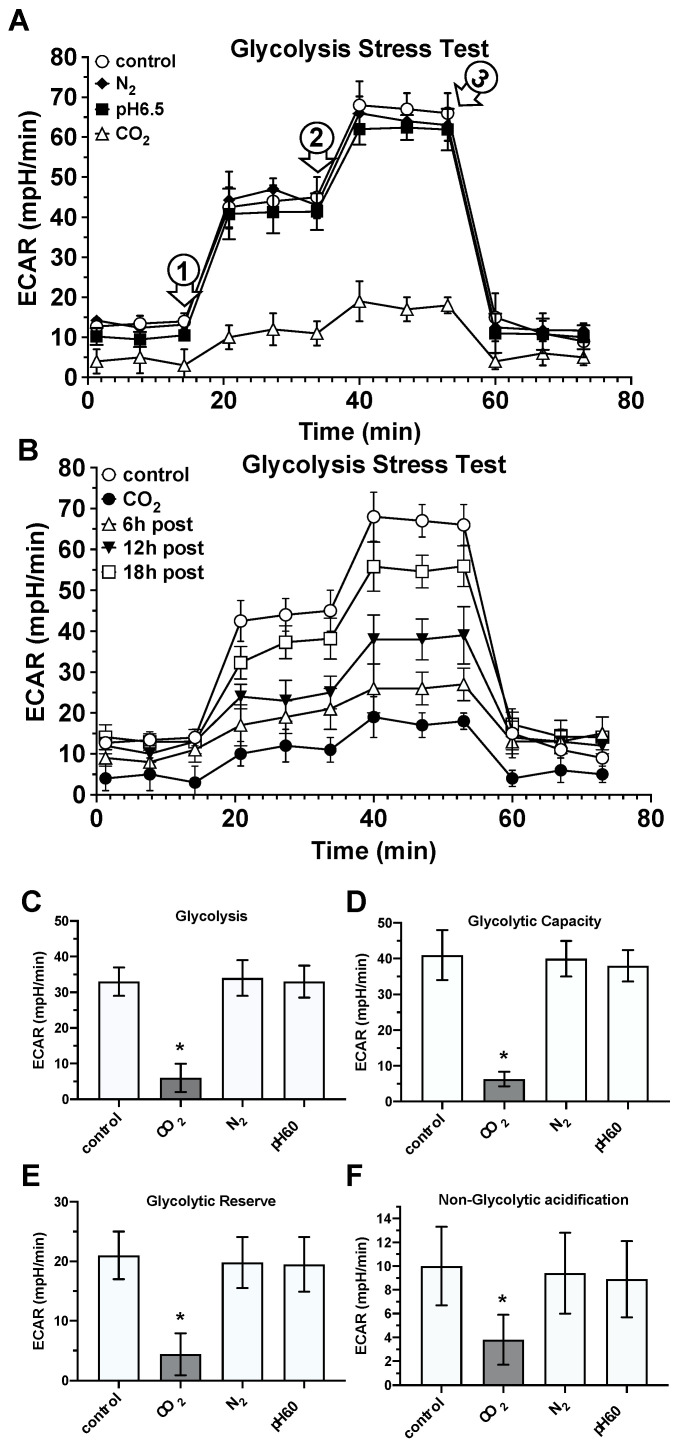
Impact of CO_2_-saturated, N_2_-saturated, and acidic solutions on glycolysis parameters. By using the Glycolysis Stress Test and the extracellular flow analyzer Agilent Seahorse XF24, the extracellular acidification rates (ECARs) were recorded in the control cultures (white circles) and CO_2_- (white triangles), N_2_- (black diamonds), or acidic media (pH 6.5; black squares)-exposed fibroblast cultures a short time after treatment (**A**) or as shown in (**B**), where ECAR was recorded in CO_2_-exposed fibroblast cultures 6, 12, or 18 h after the exposure. (**C**–**F**), the calculated values of glycolysis-specific parameters, (**C**) basal glycolysis, (**D**) glycolytic capacity, (**E**) glycolytic reserve, and (**F**) non-glycolytic acidification rate, were examined. The numbered arrows in (**A**) indicate the time points of the addition of the respective test substances: 1, oligomycin; 2, FCCP; 3, rotenone and antimycin A. Bars represent the mean ± S.D. of six individual experiments (*n* = 6) with the control cultures (white bars) and cultures exposed for 30 min to CO_2_- or N_2_-saturated solutions or to pH 6.5. *, *p* < 0.05 as compared to the control, N_2_- or pH 6.0-exposed cell cultures.

***Glycolysis:*** By recording the extracellular acidification rates (ECARs) by using the extracellular flow analyzer *Agilent Seahorse XF24*, we analyzed the impact of CO_2_- or N_2_-saturated solutions, acidified medium (pH 6.0), and lactate on several energy production-relevant parameters of glycolysis, i.e., basal glycolysis, glycolytic reserve, and capacity as well as the non-glycolytic acidification rate. As shown in Figure 8, treating the cells with CO_2_-saturated solutions resulted in a significant reduction in ECAR values correlating with the parameters mentioned above. This reducing effect could only be achieved with the CO_2_-saturated solutions, but not with the N_2_-saturated solutions or acidified medium. Interestingly, lactate-containing media exerted analogous inhibitory effects on the listed parameters of glycolysis as we observed after incubation with CO_2_-containing media.

**Figure 9:**
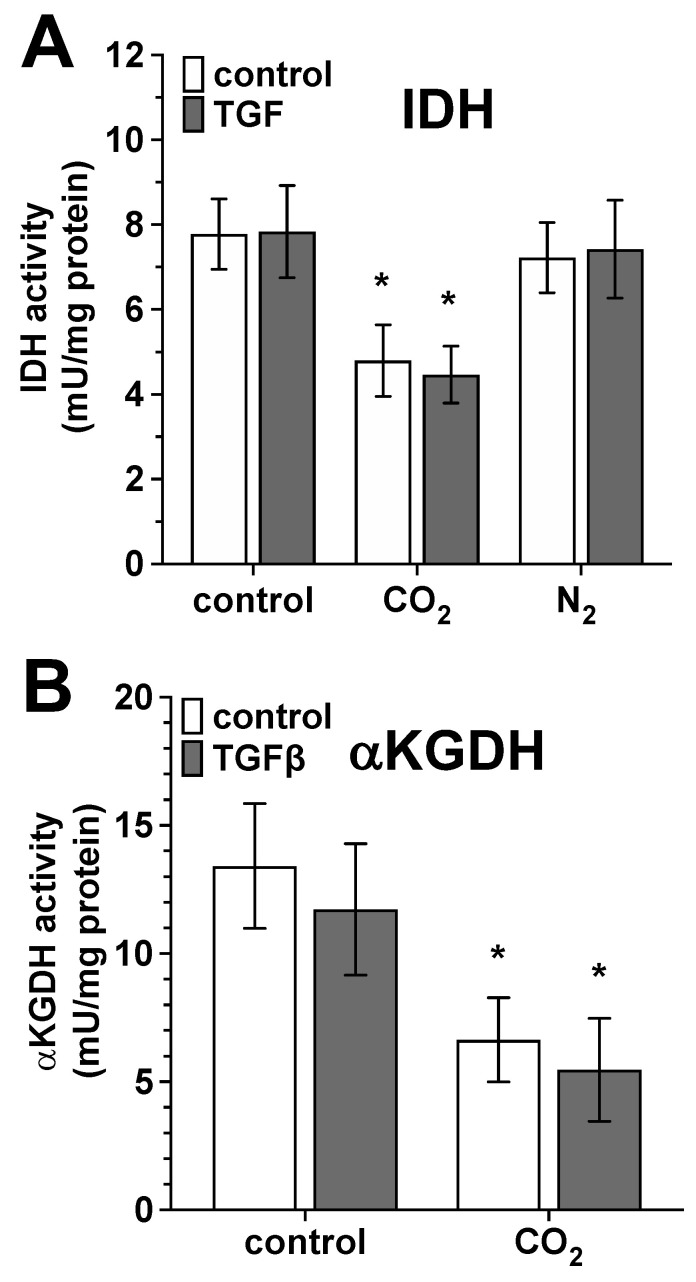
Impact of CO_2_- and N_2_-saturated solutions on the IDH2 and aKGDH activity of primary human skin fibroblasts. The untreated (white bars) and TGF-β-activated (gray bars) fibroblast cultures were treated with CO_2_- or N_2_-saturated solutions for 30 min per day during the three-day differentiation phase. At the end of the differentiation phase, the activity of iso-dehydrogenase 2 ((**A**), IDH2) and alpha-ketoglutarate dehydrogenase ((**B**), aKGDH) was quantified. The values represent the mean ± S.D. of four individual experiments (*n* = 4). In (**A**), *, *p* < 0.05 as compared to the control or N_2_-exposed cell cultures. In (**B**), *, *p* < 0.05 as compared to the control cell cultures.

***Citric acid cycle:*** The TGF-β-activated fibroblast cultures were treated with CO_2_- or N_2_-saturated solutions for 30 min per day during the three-day differentiation phase. At the end of the differentiation phase, we detected the activity of IDH2 and aKGDH, two relevant key enzymes of the citric acid cycle. Treating the untreated and TGF-β-activated cells with CO_2_-saturated solutions led to a significant reduction in IDH activity to the same extent (Figure 9A). This effect appeared to be CO_2_-specific, as the sham treatment of the cells with N_2_-saturated solutions did not induce a statistically significant change in IDH activity. We observed analogous CO_2_-induced effects regarding the activity of aKGDH, which was also statistically significantly reduced by CO_2_ treatment in its activity (Figure 9B).

**Figure 10:**
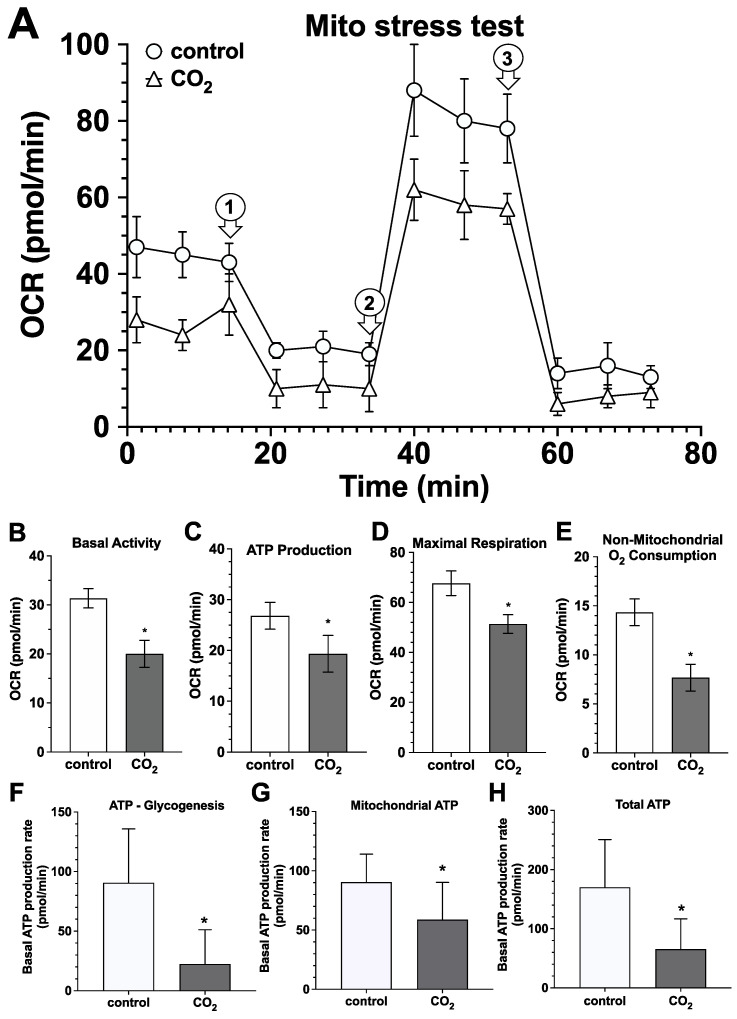
Impact of CO_2_-saturated solutions on the parameters of mitochondrial respiration and ATP production rates. (**A**), Using the Mito Stress Test and the extracellular flow analyzer Agilent Seahorse XF24, oxygen consumption rates (OCRs) were recorded in the control (white circles) and CO_2_-treated hsFB cultures (white triangles). The numbered arrows indicate the time points of the addition of the respective test substances: 1, glucose; 2, oligomycin; 3, 2-DG. The values represent the mean ± S.D. of six individual experiments (*n* = 6) with the control cultures (white circles) and cultures exposed for 30 min to CO_2_ (white triangles). The values of the glycolytic stress were used for the calculation of the values shown in (**B**), basal activity; (**C**), ATP production; (**D**), maximal respiration; (**E**), non-mitochondrial O_2_ consumption. Bars represent the mean ± S.D. of six individual experiments (*n* = 6) with control cultures (white bars) and cultures exposed for 30 min to CO_2_ (gray bars) prior to the test. *, *p* < 0.05 as compared to the control cell cultures. Additionally, we examined the impact of CO_2_-saturated solutions on ATP production rates by recording the proton efflux rates by the Agilent analyzer which allowed us to calculate glycolytic (**F**), mitochondrial (**G**), and total ATP production rates (**H**) in the control (white bars) and CO_2_-treated hsFB cultures (gray bars). Bars represent the mean ± S.D. of six individual experiments (*n* = 6) with the control cultures (white bars) and cultures exposed for 30 min to CO_2_ (gray bars) prior to the test. *, *p* < 0.05 as compared to the control cell cultures.

**Mitochondrial respiration and ATP production rate:** By recording the oxygen consumption rates (OCRs) by using the extracellular flow analyzer, we analyzed the impact of CO_2_ on several energy production-relevant parameters of mitochondrial respiration, i.e., basal activity, ATP production, maximal respiration, and non-mitochondrial O_2_ consumption. We show in Figure 10A–E that the exposure of the hsFB cultures to CO_2_-saturated solutions for 30 min statistically significantly reduced the values of the examined parameter of mitochondrial respiration.

Additionally, we have recorded the proton efflux rate (PER) by the Agilent analyzer (Figure 10F–H) and found a strong and statistically significant reduction in glycolytic, mitochondrial, and therefore, also in the total ATP production rates in CO_2_-exposed hsFB cultures.

### 2.5. CO_2_ Exposure Enhanced Local Blood Flow and Tissue Oxygen Saturation in Human Skin Tissue of the Hand In Vivo

In order to demonstrate that CO_2_ can diffuse deeply into human skin tissue and induce a biological response after a 15-min immersion of the hand in CO_2_-saturated water (20 °C), we evaluated blood flow and tissue oxygen saturation in skin layers 1–4 mm deep using the O2C device and the Kent camera before and after CO_2_ exposure.

**Figure 11:**
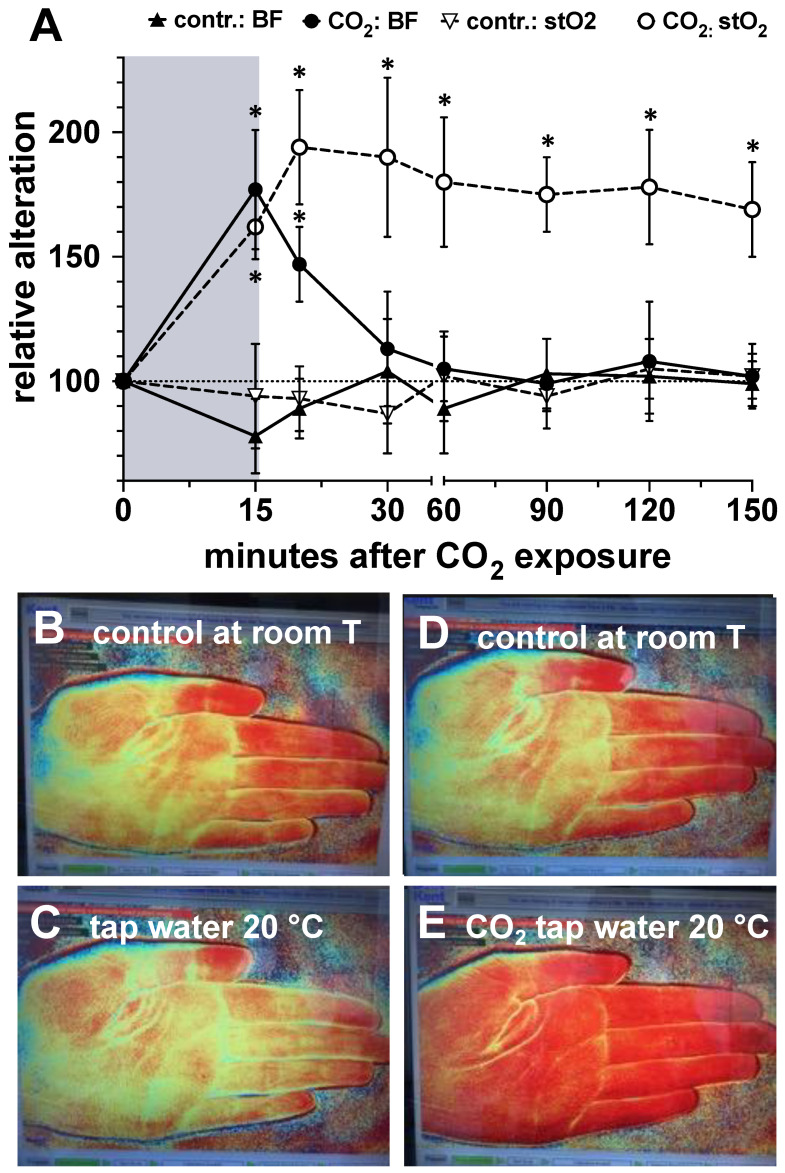
CO_2_-mediated modulation of local blood flow and tissue oxygen saturation in human skin tissue in vivo. (**A**), Relative alteration of blood flow and tissue oxygen saturation at a depth of 1 to 4 mm in the palmar region of the hand as detected by the O2C device prior to or after a 15 min bath (gray area) of the hand in natural or CO_2_-saturated tap water at 20 °C (▲, blood flow (BF) in tap water at 20 °C; ●, blood flow (BF) in CO_2_-saturated tap water at 20 °C; ▽, tissue O_2_ saturation (stO_2_) in tap water at 20 °C; ○, tissue O_2_ saturation (stO_2_) in CO_2_-saturated tap water at 20 °C). The respective values obtained with hands not exposed to water at all (negative control at t = 0 min) were set to 100%. *, *p* < 0.05 as compared to the control values obtained prior to water exposure. (**B**–**E**), Representative photos taken with the Kent camera showing a signal for oxidized hemoglobin in the palmar region of a control hand (red color) prior to (**B**) and after a 15 min lasting bath in 20 °C tap water (**C**) and control hand prior to (**D**) and after a 15 min lasting bath in 20 °C CO_2_-saturated tap water (**E**).

As we show in Figure 11, only the treatment of the hand in CO_2_-containing bath solutions led to a statistically significant increase in blood flow and tissue oxygen saturation in the skin. While the increase in blood flow returned to pre-exposure levels just 15 min after the end of the CO_2_ bath, the tissue oxygen saturation values remained significantly higher than the baseline levels before the CO_2_ exposure until the end of the experiment, 135 min after exposure.

## 3. Discussion

Human dermal fibroblasts are pivotal in the physiological functioning of the skin, particularly in preserving its structural integrity and orchestrating vital phases of the wound-healing process. These cells specialize in synthesizing, organizing, and remodeling the extracellular matrix (ECM) [31,32]. During wound repair, fibroblasts undergo a critical transformation into myofibroblasts, a process central to wound granulation, tissue contraction, and ultimately, wound closure. Disruption in these stages of wound healing, characterized by the enhanced proliferation, activity, or differentiation of fibroblasts, plays a critical role in pathological conditions such as scleroderma, hypertrophic scars, and keloids. Myofibroblasts, known for their contractile abilities, are predominantly identified by the presence of α-smooth muscle actin (α-SMA) [33,34]. The key drivers of fibroblast differentiation include the growth factor TGF-β1, which fibroblasts can also produce, and mechanical stimuli like tension. Myofibroblast differentiation involves multiple differentiation stages, beginning with the transformation into α-SMA-negative proto-myofibroblasts, which can generate stress fibers containing beta and gamma cytoplasmic actin. This is followed by their final evolution into α-SMA-positive myofibroblasts. Typically, these cells are eliminated from the healing tissue through apoptosis once repair and wound closure are completed [35,36]. Unrestrained collagen production and the contractile activities of myofibroblasts are primary contributors to the development of the conditions mentioned earlier [37,38]. Fibrotic disorders present considerable therapeutic challenges due to their intricate pathophysiology. Despite progress in deciphering the underlying biological mechanisms, effective treatments for fibrosis remain elusive. Furthermore, there is still some controversy in the medical community regarding the best standard treatment for abnormal scar management [39].

In the present study, we aimed to investigate the potential therapeutic effects of exogenously applied carbon dioxide (CO_2_) on fibroblast behavior, focusing on viability, proliferation, migration, and myofibroblast differentiation. The main finding of our study is the significant reduction in α-SMA and EDA-fibronectin protein expression, both representing relevant markers of myofibroblast differentiation, following exposure to CO_2_ in solutions. This reduction was observed even with short exposure durations, such as one minute per day, suggesting a rapid and potent inhibitory effect of CO_2_ on myofibroblast differentiation. In the experiments with CO_2_-saturated solutions, we must assume that a portion of the CO_2_ diffused out of the medium during the course of the experiment, probably resulting in a lower CO_2_ concentration in the solutions after just a few minutes compared to the beginning of the experiment. However, regardless of this, we achieved identical results in terms of the inhibition of myofibroblast differentiation with the CO_2_-containing solutions as with the cultures treated with CO_2_ in the hyperbaric chamber, which were exposed to maximum CO_2_ saturation throughout the entire experimental period. Importantly, this effect was specific to CO_2_ exposure, as exposure to nitrogen (N_2_)-saturated solutions did not induce statistically significant changes in α-SMA expression. Furthermore, we found that the degree of reduction in α-SMA expression correlated with the level of CO_2_ saturation of the solutions, indicating a dose-dependent response. These findings suggest that CO_2_ may possess antifibrotic properties by attenuating myofibroblast differentiation, a critical process in fibrotic tissue remodeling. Furthermore, we would like to point out our further findings that under the experimental conditions used here, exposure to CO_2_ or N_2_, despite the induced hypoxia, did not lead to any statistically significant changes in relation to the vitality, proliferation, or migration of human skin fibroblast cultures. These results represent highly interesting findings as, due to the high diffusion constant of CO_2_ and due to its greater solubility in water and biological fluids than O_2_ [40,41], carbon dioxide, as we also show here, can rapidly and deeply penetrate into exposed tissues in vivo. Due to its apparent fibroblast differentiation-reducing properties, the use of exogenously applied CO_2_ could represent a cost-effective, low side-effect, and effective local therapy option as a prophylaxis against the fibrotic and sclerotic complications of the skin tissue.

It is difficult to contextualize our observations within the existing literature as despite intensive research, except for one publication, we have been unable to locate any studies in the scientific databases that address the topic of the effect of exogenously applied CO_2_ on myofibroblast differentiation. Our findings, which to our knowledge are the first to suggest a pharmacological-like response of fibroblasts to exogenously applied CO_2_, are consistent with the results of a study by Tadokoro et al. that has focused on the impact of CO_2_ on the expression of TGF-β and α-SMA of cancer-associated fibroblasts in a mouse model in vitro. In that study, CO_2_ was applied in the form of a CO_2_-containing hydrogel for 20 min. Regardless of the differences in experimental setup, Tadokoro et al. also observed a significant CO_2_-induced reduction in α-SMA protein expression and other fibroblast markers, which were exclusively evaluated by immunohistochemical methods [42].

One of the first considerations when pondering the underlying mechanism of the modulation of TGF-β-induced myofibroblast differentiation by CO_2_ is the issue of pH. CO_2_ affects the pH of the extracellular environment by forming carbonic acid (H_2_CO_3_) when reacting with water, leading to acidification. In the case of CO_2_-saturated solutions, as we show here in Figure 6, this led to a reduction to approximately pH 6.5. And even using appropriate biologically compatible buffer systems, the pH stability of the carbonized solutions could not be achieved. Changes in pH can modulate the activity of various enzymes and signaling pathways that might also be relevant in fibroblast differentiation (for review see [43]). Due to the numerous potential effects of decreased pH on cellular physiology, we have devoted significant experimental attention to this topic. Based on our results from the various control experiments, in our in vitro experimental setup we can confidently exclude the possibility that the observed anti-myofibrotic effects of CO_2_ can be solely explained by a decreased pH.

Another molecular factor behind the described myofibroblast differentiation-reducing effect of CO_2_ could be hypoxia and an increase in the stability or expression of HIF-1α. In our study, however, there are legitimate doubts about hypoxia as the underlying mechanism, especially as also with an N_2_-saturated solution, no evidence of an associated reduction in α-SMA expression could be observed. But also independently of this consideration, hypoxia and increased HIF-1α protein expression are primarily associated with profibrotic and not antifibrotic activity (for review see [44]). In several models of renal and hepatic fibrosis and other fibrotic complications, HIF-1α signaling pathways seem to play an active role in promoting fibrosis [45,46,47,48,49,50]. The overexpression of HIF-1α in human skin has also been linked to the progression of fibrotic diseases, as it promotes myofibroblast differentiation and leads to excessive matrix production and deposition [51,52,53,54,55]. It should be noted at this point, however, that there are also findings suggesting an opposite role of hypoxia, indicating that hypoxia can impair the differentiation and function of skin myofibroblasts [40].

The profibrotic factor TGF-β enhances glycolysis in dermal fibroblasts [56,57]. Numerous experimental studies have established a strong causal link between aerobic glycolysis and the activation and differentiation of myofibroblasts [58]. Aerobic glycolysis simultaneously supports both the differentiation and contractile functions of myofibroblasts driven by TGF-β [58,59,60]. Alterations in glycolysis are crucial not only for energy production but also for regulating the related biosynthetic pathways that provide the necessary components for cellular growth and differentiation [61]. The first indication of “glycolytic remodeling” in activated fibroblasts was found in cystic fibrosis patients, where an increased glycolysis and glucose metabolism enzyme activity was observed [61]. A glycolytic shift in fibroblasts has since been confirmed in several fibroblast populations [62,63]. One way to boost glycolysis is through the transcriptional upregulation of various glycolytic genes [62,64,65], many of which act as metabolic checkpoints. As such, glycolytic reprogramming represents a crucial metabolic change that fosters myofibroblast differentiation and fibrotic progression, suggesting that targeting glycolytic metabolism could be a promising therapeutic approach for treating fibrotic diseases [58,66]. Notably, inhibiting glucose uptake and glycolysis with 2-deoxyglucose (2DG) prevents α-SMA expression [62,65]. 2DG, a glucose analog, acts as a non-competitive inhibitor of hexokinase [67,68,69], and by inhibiting the initial key steps in glucose metabolism, glycolysis can be partially disrupted [70]. Therefore, blocking glucose metabolism in fibroblasts might be an effective strategy to interfere with profibrotic pathways [65], as this ultimately results in reduced ATP production, cell cycle arrest, inhibited cell growth, and even cell death [71,72]. In fact, we achieved the complete inhibition of TGF-β-induced increase in α-SMA expression in human skin fibroblasts by using 2DG at a non-toxic concentration. This process was accompanied by a significant reduction in the glycolytic and mitochondrial ATP production rates. Against this background and also due to the following findings, we, therefore, postulate that the molecular mechanism behind the CO_2_-induced inhibition of TGF-β-induced myofibroblast differentiation might be based on a CO_2_-specific inhibition and reduction in glycolysis and/or mitochondrial respiration and probably a resulting breakdown in cellular ATP production.

Indeed, we found that CO_2_ exposure of the fibroblast cultures led to an almost complete inhibition of glycolysis and, nterestingly, it took more than 18 h before the values of reduced glycolysis returned to the levels of the untreated control cell cultures. It is important to mention that in order to rule out direct pH effects, the glycolysis assay was carried out after several medium changes approximately one hour after the CO_2_ exposure. In addition, we observed energy metabolism-relevant effects of CO_2_ on the level of the citric acid cycle and mitochondrial respiration. Here, we found that CO_2_ treatment significantly reduced the activity of isocitrate dehydrogenase 2 (IDH2) and α-ketoglutarate dehydrogenase (αKGDH), two key enzymes of the citric acid cycle, but also led to a significant reduction in the key parameters of mitochondrial respiration. The last-described effects ultimately led to a highly significant reduction in the total cellular ATP production rate. These findings highlight the intricate interplay between CO_2_ exposure and cellular metabolism, implicating CO_2_ as a modulator of metabolic homeostasis in fibroblasts. These findings are consistent with the abovementioned concept of glycolytic reprogramming in myofibroblast differentiation by Xie et al. [65] as well as previous findings of Vohwinkel et al., who showed that patho-physiologically increased CO_2_ levels can lead to mitochondrial dysfunction due to a reduction in IDH2 and αKGDH activity [23].

As we also demonstrate in this study, CO_2_ diffuses rapidly into exposed tissue in vivo and induces corresponding local biological responses, such as an increased local blood flow and, in parallel, a significant increase in the level of tissue oxygen saturation (stO_2_). However, one further observation requires particular attention. In the in vivo experiment, as expected, the increase in local blood flow returned to baseline within a comparatively short time interval of approx. 15 min after CO_2_ exposure. However, the stO_2_ of the tissue remained significantly elevated for more than two hours after CO_2_ exposure. While it can be assumed that the initial increase in stO_2_ was a function of the elevated blood flow, the prolonged elevation of stO_2_ must be attributed to a different mechanism. If the increase in stO_2_ is not a function of blood flow, it can only be the result of an increased O_2_ accumulation due to reduced O_2_ consumption by the cells in the tissue. This assumption would be fully consistent with our in vitro experiments and the observation of CO_2_-reduced ATP metabolism.

In conclusion, exogenously applied CO_2_ can directly reduce α-SMA expression and myofibroblast development, a crucial step for the deposition of the extracellular matrix and tissue scarring seen in fibrosis. Furthermore, the impact of CO_2_ on cellular metabolism, specifically its inhibition of glycolysis, mitochondrial respiration, and the key enzymes of the citric acid cycle, provides additional insights into the molecular mechanisms of its potential antifibrotic effects. Therefore, exogenously applied CO_2_ could serve as a novel antifibrotic agent by leveraging mechanisms similar to the established treatments but through a distinct and possibly more directly controllable modality. Its ability to simultaneously inhibit critical protein expressions involved in fibroblast function and severely restrict metabolic pathways offers a dual mechanism of action that could be highly effective in the prevention of fibrotic skin diseases.

## 4. Materials and Methods

### 4.1. Materials

If not otherwise indicated, all the chemicals, antibodies, and assay kits were purchased from Sigma-Aldrich Chemie GmbH (Munich, Germany).

### 4.2. Cell Culture

Human skin fibroblasts (hsFBs) were isolated from human skin specimens obtained with patients’ consent from eight female and four male patients aged between 20 and 65 years who underwent plastic breast or abdominal surgery and taken in cell culture under identical conditions as described previously [12]. Briefly, skin samples maintained in a Petri dish were cut into small pieces and were incubated in Dispase II solution overnight at 4 °C. The next day, epidermis and dermis were separated and the fibroblast-containing dermis was incubated with collagenase for 60 min at 37 °C in a tempered shaker, and then vortexed and passed through a sieve (50–100 µM) to drain the fibroblasts from the samples. After a centrifugation step the supernatant was removed, and the cell pellet resuspended with 10 mL of new DMEM. Finally, the isolated fibroblasts were given into cell culture flasks and put into the incubator (37 °C, 5% CO_2_) to grow.

The experimental protocol and the use of human material have been approved by the local Ethics Committee of the Medical Faculty of the Heinrich-Heine-University Düsseldorf (study number: 3634) and are in accordance with the Declaration of Helsinki.

### 4.3. Generation of CO_2_- or N_2_-Saturated Solutions

The fibroblasts were treated with CO_2_ in two fundamentally different ways. Firstly, with CO_2_-saturated media, or alternatively, in a pressurized chamber flooded with CO_2_ at an overpressure of 2 bar (29.4 PSI). While in the first case, the CO_2_ concentration of the solutions used decreased over time due to diffusion, in accordance with the gas laws and temperature dependence, the CO_2_ concentration in the cell culture supernatants of the cultures treated in the hyperbaric chamber remained constant throughout the entire exposure period.

CO_2_-saturated solutions were prepared by using the medical device CARBOTHERA K104 from Mitsubishi Rayon Cleansui (Mitsubishi Rayon Cleansui Co., Ltd., Tokyo, Japan). In this case, Dulbecco’s Modified Eagle Medium (DMEM) without fetal bovine serum (FBS) was sucked from a container into the device and was transferred there under pressure through a network of membrane capillaries, wherein CO_2_ gas, which was provided from a CO_2_ gas cylinder, was forced through the membrane in the liquid. The freshly prepared CO_2_-saturated DMEM solution (DMEM-CO_2)_, containing 1100–1200 mg CO_2/_1000 mL (pH 6.6), was then used for the experiments.

Alternatively, we produced CO_2_- or N_2_-saturated solutions using an experimental hyperbaric laboratory test chamber that can be filled with appropriate gasses (HAUX Testcom 350/10, HAUX-Life-Support GmbH, Karlsbad-Ittersbach, Germany) at an overpressure of 2 bar.

### 4.4. Induction of Fibroblast Differentiation and CO_2_ Treatment Protocols

The effect of CO_2_ on fibroblast differentiation was examined with TGF-β-activated fibroblasts, which differentiate into myofibroblasts, a cell type that is characterized by the expression of alpha-smooth muscle actin (α-SMA), a contractile structure that serves as a reliable marker of fibroblast differentiation and fibronectin extracellular domain (EDA-FN).

On day 1, confluent cell layers with human skin fibroblast growing in 24-well cell culture plates were treated with the freshly prepared DMEM-CO_2_ for the time intervals indicated (1, 2.5, 5, 15, or 60 min). Then, DMEM-CO_2_ was replaced by a fibroblast culture medium and the fibroblast cultures were additionally incubated for 24 h in 5 ng/mL TGF-β. This procedure was repeated on days 2 and 3. Alternatively, we have incubated fibroblast cultures for 15 or 60 min in DMEM-diluted DMEM-CO_2_ which contained 75%, 50%, 25%, 12%, 6%, or 3% of the CO_2_ of the original DMEM-CO_2_ content. At day 4, the cells were lysed by RIPA-lysis buffer and harvested by cell-scraping and the material was used for a Western blot analysis. Alternatively, the experiments mentioned above were carried out with N_2_-saturated solutions instead of CO_2_-saturated solutions as a specificity control.

### 4.5. Toxicity Assay

Human skin fibroblasts growing to confluency on the bottom of cell culture plates (24-well) were incubated with freshly prepared DMEM-CO_2_ for 5, 10, 15, 30, and 60 min. Then, 3 h, 6 h, and 24 h after CO_2_ exposure, viability was measured using the CellTiter-Blue cell viability assay (Promega Corporation, Madison, WI, USA) as described previously [73,74]. Cell viability and the appearance of necrotic and apoptotic cell morphology were investigated by fluorescence microscopy and using the fluorescence dyes fluorescein diacetate, Hoechst 33342, and propidium iodide (each dye 0.5 µg/mL) [75,76], whereby the effect of CO_2_ exposure on cell death via apoptosis additionally was examined by a FACS analysis by the method described by Riccardi et al. [8] 3 and 24 h after exposure. Staurosporine at a concentration of 1 µg/mL was used as a positive control. Alternatively, the experiments mentioned above were carried out with N_2_-saturated solutions instead of CO_2_-saturated solutions as a specificity control.

### 4.6. Proliferation Assay

Fibroblasts (1 × 10^4^ cells grown in 24-well cell culture plates) were incubated on days 1, 2, and 3 after adherence for 5, 10, 15, 30, or 60 min with DMEM-CO_2_. On day 4, the cell number of the respective cultures was detected by the CellTiter-Blue (Promega Corporation, Madison, WI, USA) assay as described above.

### 4.7. Fibroblast Migration Assay

The impact of CO_2_ exposure on the migration of fibroblasts was evaluated using a scratch assay [77]. Here, we have defined the area of the scratch as 100% and have evaluated the remaining cell-free area 24 h after one 60 min lasting exposure of the cell cultures to CO_2_-saturated solutions, given in %.

### 4.8. Immunocytochemistry

In order to visualize α-SMA-positive myofibroblast, TGF-β-treated hsFB cultures maintained in 6-well cell culture plates were fixed by 15 min treatment with 4% paraformaldehyde/PBS and permeabilized by 0.2% Triton X-100/PBS. The cell samples were incubated with blocking buffer (4% BSA/PBS) for 30 min and subsequently with a monoclonal mouse anti-human α-smooth muscle actin (α-SMA, Abcam, Cambridge, UK) antibody diluted 1:400 in blocking buffer for 60 min. α-SMA-staining was visualized using the Dako REAL^TM^ detection system (Alkaline Phosphatase/RED, Rabbit/Mouse, Code K5005; Agilent, Santa Clara, CA, USA) and the Axiovert 200 fluorescence microscope and the integrated Axiocam MRc camera (Carl Zeiss AG, Jena, Germany).

### 4.9. Cell Protein Collection, Gel Electrophoresis, Western Blotting, and Real-Time qPCR

Cell protein collection, gel electrophoresis, and Western blotting were performed exactly as described previously [73,74]. The Western blot analysis of protein expression was performed using extracellular domain A fibronectin (EDA-FN)/ab6328 (Abcam, Cambridge, UK), GAPDH/IMG-6665A (Novus Biologicals, Cambridge, UK), and α-smooth muscle actin (α-SMA)/ab7817 (Abcam, Cambridge, UK) mouse anti-human antibodies as the primary antibodies, and as the secondary antibodies, we used goat anti-mouse IgG/HRP #P0447 or goat anti-rabbit IgG/HRP #P0449 antibodies using a 1:1000 dilution (Agilent Technologies, Santa Clara, CA, USA), respectively. Protein-specific signals detected on the nitrocellulose membrane after the immune reaction were normalized to the expression of the GAPDH protein or to the values of the total amount of proteins of the same sample detected on the nitrocellulose membrane after the blotting process and prior to the immune reaction.

For real-time qPCR, total cell RNA was isolated after lysing fibroblasts, maintained in 6-well plates, using the RNeasy^®^ Mini Kits (QIAGEN N.V., Hilden, Germany). The RNA was converted into cDNA using the Omniscript^®^ RT Kits (QIAGEN N.V., Hilden, Germany). The RT-qPCR was performed in triplicates using the Power SYBR^®^ Green PCR Master Mix (Thermo Fisher Scientific GmbH, Karlsruhe, Germany) with 1 ng cDNA for EDA-FN in a 25 µL PCR reaction with a 7300 Real-Time PCR System (Applied Biosystems, Waltham, MA, USA). For the determination of mRNA expression, primer concentrations of 0.2 µM were used. Transferrin receptor 1 (TFRC) was used as the reference gene for normalization. The forward and reverse primer sequences are listed in Table 1. The 2^−ΔΔCt^ method was employed for the calculation of relative mRNA expression.

### 4.10. Evaluation of Oxygen Consumption Rate (OCR) and Extracellular Acidification Rate (ECAR)

Oxygen consumption rate (OCR), proton efflux rate (PER), and extracellular acidification rate (ECAR) allow conclusions about different energy production-relevant parameters of mitochondrial respiration, glycolysis, and ATP production rates. ECAR, PER, or OCR was recorded by using the extracellular flow analyzer Agilent Seahorse XF24 (Seahorse Bioscience, North Billerica, MA, USA) [78,79]. The assays were performed with untreated hsFB cell cultures or with cell cultures that were exposed for 15 min to CO_2_- or N_2_-saturated solutions or to acidic solutions with pH 6.0. Additionally, assays were performed with untreated hsFB cell cultures maintained for 2 h in lactate- (5 mM) or 2-deoxy-glucose (2-DG, 6.25–50 mM)-containing media. One hour before performing the measurement with the Seahorse device, the cell culture supernatants containing the different additives mentioned above were aspirated, the cell cultures were washed twice with medium and mixed with the corresponding assay media hsFB cell cultures in accordance with the manufacturer’s recommendation and exactly as described by us recently [73].

### 4.11. Inhibition of Glycolysis by 2-Deoxy-Glucose

As a test substance to demonstrate an antifibrotic effect by inhibiting cellular energy metabolism, we used the competitive hexokinase inhibitor 2-deoxy-glucose (2-DG) [67,68,69], which inhibits the first critical steps of glucose metabolism and partially disrupts glycolysis [70]. 2-DG was used at concentrations of 6.25–50 mM and was present in the cell cultures for the time intervals indicated and its impact on glycolysis or ATP production rates was achieved by using the Agilent Seahorse XF24 analyzer.

### 4.12. Enzyme Activity Assays

The enzyme activity of Isocitrate dehydrogenase 2 (IDH2) was quantified in untreated and CO_2_- or N_2_-exposed hsFB cell cultures by using the semi-quantitative colorimetric IDH2 (R140Q) Activity Assay Kit from BPS Bioscience, (San Diego, CA, USA). The assay is designed to measure IDH2 activity by measuring NADPH consumption. The activity of a-ketoglutarate dehydrogenase (KGDH) was quantified in untreated and CO_2_- or N_2_-exposed hsFB cell cultures with the help of the semi-quantitative colorimetric a-Ketoglutarate Dehydrogenase Activity Assay Kit from Abcam (Cambridge, UK). This assay is designed to measure KGDH activity by measuring NAD consumption. Both assays were carried out exactly in accordance with the manufacturer’s recommendation.

### 4.13. Impact of CO_2_ Exposure on Local Blood Flow and Tissue Oxygen Saturation in Human Skin Tissue In Vivo

Carbon dioxide is known to induce vasodilation in various tissues, including the skin. The underlying mechanisms of CO_2_-induced vasodilation involve several biochemical pathways and cellular responses [80,81,82,83]. So, an increase in local blood flow induced by CO_2_ provides direct evidence of CO_2_ diffusion into the exposed tissue. As part of an in vivo study with healthy subjects, we used the O2C device (LEA Medizintechnik GmbH, Giessen, Germany), a combined photo spectrometer/laser Doppler flowmeter, to capture the impact of CO_2_-saturated solution (tap water, 20 °C) on changes in blood flow and tissue oxygen saturation at a depth of 1 to 4 mm exactly as described previously [84]. Additionally, we visualized CO_2_-induced effects on local blood flow by using a multispectral camera from Kent (Kent Imaging Inc., Calgary, AB, Canada).

In this blinded, two-armed, and randomized study, five healthy subjects were included. Over several sessions, they immersed one hand in a CO_2_-saturated bath or in a non-carbonated bath. Measurements were taken prior, immediately after, and at the time points indicated after the respective bath procedure. The study was performed after collecting information and written participants’ consent for the study. The experimental protocol has been approved by the local Ethics Committee of the Medical Faculty of the Heinrich-Heine-University Düsseldorf (study number 5806R, registration-ID 2017044214) and is in accordance with the Declaration of Helsinki.

### 4.14. Statistical Analysis

For statistical analysis, we used GraphPad Prism 8 (San Diego, CA, USA). Significant differences were evaluated using 1-way ANOVA followed by an appropriate post hoc multiple comparison test (Tukey method) or the Wilcoxon test or Student’s *t*-test. A *p*-value of <0.05 was considered significant.

## Figures and Tables

**Table 1:** List of RT-qPCR primers.

Target Gene	Forward Primer (5′ → 3′)	Reverse Primer (5′ → 3′)	Product Length
EDA-FN	ACTGATTGCACTTCTGAGGGCAG	GATTTCCTCGTGGGCAGCCA	112
TFRC	TTCAGGTCAAAGACAGCGCTCA	CTATACGCCACATAACCCCCAGG	100
α-SMA	AGCCAAGCACTGTCAGGAAT	TTGTCACACACCAAGGCAGT	108

## Data Availability

The datasets used and/or analyzed during the current study are available from the corresponding author upon reasonable request.
